# High Outcome-Reporting Bias in Randomized-Controlled Trials of Acupuncture for Cancer Chemotherapy-Induced Nausea and Vomiting: A Systematic Review and Meta-Epidemiological Study

**DOI:** 10.3390/curroncol32080462

**Published:** 2025-08-15

**Authors:** Rachele Penati, Riccardo Vecchio, Roberto Gatto, Anna Odone, Silvia Deandrea

**Affiliations:** 1 Villa Beretta Rehabilitation Center, Valduce Hospital, 23845 Costa Masnaga, Italy; 2 Centro Studi So Wen ETS, 20146 Milano, Italy; 3 Department of Public Health, Experimental and Forensic Medicine, University of Pavia, 27100 Pavia, Italy; 4 Medical Direction, Fondazione IRCCS Policlinico San Matteo, 27100 Pavia, Italy

**Keywords:** bias, complementary therapies, nausea, vomiting, chemotherapy, acupuncture therapy

## Abstract

Selective outcome-reporting bias refers to the selective reporting of a subset of study findings. This methodological limitation may occur in cancer-related acupuncture studies, where valid empirical studies on psychometric performance are still lacking. We assessed the risk of selective outcome reporting bias in studies published in English that were included in a systematic review on acupuncture for preventing cancer chemotherapy-induced nausea and vomiting. For each study, we searched for registry availability and, if present, assessed its validity. We described each study outcome (nausea, vomiting, or both) according to the following seven items: type of outcome, domain, specific measurement, specific metric, type of data, methods of aggregation, and timepoint unit and time. Eleven studies published between 1987 and 2019 in English were evaluated. Only four (36%) had a registry, of which only two were prospective and therefore considered valid. Discrepancies were found in the specific measurement of the outcome in two studies and in the specific metric. In many other cases, discrepancies were not evaluable due to missing information. No study reported complete outcomes as planned in the published protocol. Communication about the importance of prospective trial registration, including outcome details, should be enforced to reduce the risk of selective outcome reporting bias in oncology acupuncture studies.

## Simple Summary

Reporting all the results of scientific studies is important to make sure treatments are tested fairly and transparently. We looked at clinical studies that tested acupuncture to reduce nausea and vomiting caused by cancer treatments. We found that many of these studies did not describe their results completely or clearly, and some had not been registered in public databases before starting. Our study shows that more attention should be given to clear, complete, and early reporting of outcomes in clinical research, especially when treatments are based on patient-reported symptoms. Following these good practices can improve future studies and help doctors and patients make better-informed decisions.

## 1. Introduction

Outcome-reporting bias is a major issue in clinical research, notably with subjective outcomes or complex interventions. It involves selectively reporting certain outcomes while omitting others based on their nature, significance, or relevance to publication [1]. This bias compromises evidence-based medicine, skews meta-analyses, and can mislead clinical decisions. There are three possible types of selective outcome reporting. Firstly, some studies may report only a selection of outcomes, omitting those that do not present data deemed significant enough to enhance the likelihood of publication. Second, there could be selective reporting of a specific outcome, which occurs when an outcome is measured and analyzed at multiple timepoints but not all results are reported. Third, there could be incomplete reporting of a specific outcome, which happens when an outcome is not fully reported as planned [2]. The presence of selective outcome reporting is assessed in current risk of bias tools such as RoB2, ROBINS-I, etc. [3].

A recent study focusing on randomized controlled trials (RCTs) of Traditional Chinese Medicine (TCM) identified discrepancies in the efficacy (29%) and safety outcome (28%) between the trials’ registrations and their subsequent publications [4]. Another study [5] found that only 19.3% of the trials had been registered before the start of the trial. In addition, inconsistencies between the planned and reported primary outcomes were identified in 45.1% of the studies (71.4% of which had a discrepancy that favored statistically significant primary outcomes).

Selective outcome reporting has also been observed in oncology clinical trials, where biases in reporting primary endpoints and toxicity, omission of pre-registered outcomes, selective reporting in later trial phases, and delayed protocol registration can lead to misinterpretation of results, raising concerns about transparency in clinical research [6,7].

These biases are particularly problematic in acupuncture studies for chemotherapy-induced nausea and vomiting (CINV), where patient-reported outcomes (PROMs) such as nausea severity or vomiting frequency are often primary endpoints. Unlike pharmacological trials, where biomarkers and laboratory measures often dominate, acupuncture trials depend on subjective outcomes that are more vulnerable to bias. Moreover, many acupuncture trials originate in settings with less stringent trial registration enforcement or a lack of familiarity with detailed reporting guidelines.

These findings indicate that selective outcome reporting in TCM trials is a significant issue, complicating evidence synthesis and interpretation in the field. The aim of this meta-epidemiological study is to evaluate the selective outcome reporting in RCTs of acupuncture for preventing CINV [8]. By evaluating trial registrations (when available) and published reports against structured outcome descriptors, we aim to clarify the consistency and completeness of reporting practices. Our goal is to inform methodological improvements in future integrative oncology research, fostering transparency, reproducibility, and clinical trust.

## 2. Materials and Methods

We assessed selective outcome reporting in the RCTs published in English that were included in a systematic review of the effects of acupuncture in preventing CINV (Open Science Framework registration https://doi.org/10.17605/OSF.IO/AHCWK, accessed on 16 May 2025) [8]. This systematic review and meta-analysis evaluated the efficacy and safety of acupuncture in preventing chemotherapy-induced nausea and vomiting (CINV) among cancer patients. Researchers conducted comprehensive searches across eight databases, including MEDLINE, EMBASE, Cochrane CENTRAL, CINAHL, and several Chinese databases (Chinese Biomedical Literature Database, VIP, CNKI, Wanfang), to identify randomized controlled trials (RCTs) comparing acupuncture with sham acupuncture or usual care. A combination of relevant controlled vocabulary (MeSH—Medical Subject Headings in PubMed) and text/free word was used (Supplementary File). All studies from inception until June 2020 were identified. No date restrictions were applied. We included only studies in English language.

The primary outcome assessed was the complete control of CINV, defined as no vomiting episodes and/or no more than mild nausea. The certainty of evidence was appraised using the GRADE approach according to risk of bias, consistency, indirectness, and imprecision. Sources of bias assessed were as follows: random sequence allocation, allocation concealment, performance bias, detection bias, attrition bias, and selective outcome reporting [8]. The latter source of bias is the focus of the analysis we performed.

Therefore, to assess RCTs selective outcome reporting, we created a template based on the five-item definition of outcomes by Saldanha et al. [9]: (1) domain; (2) specific measurement; (3) specific metric; (4) method of aggregation; and (5) timepoint. We piloted the data extraction template on four studies. For the remaining studies, one person (RP) performed the assessments, and another one (SD) crosschecked the extracted data.

**Figure 1:**
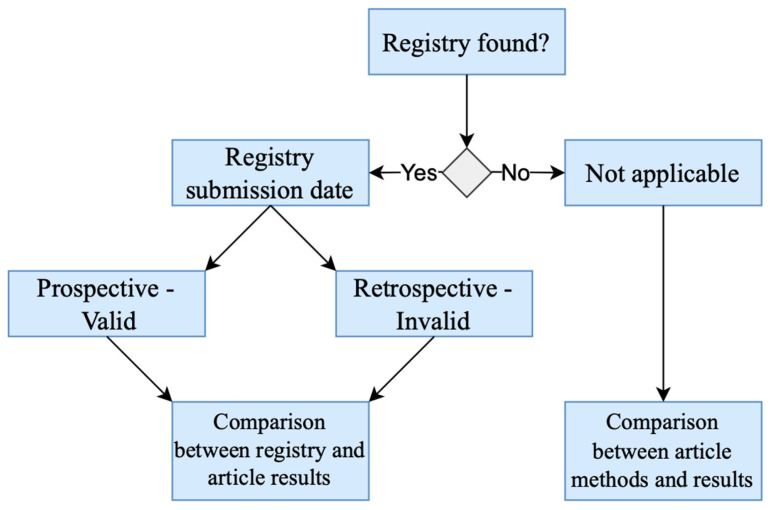
Process of definition and selection of sources for outcome reporting bias assessment.

Firstly, we identified the original source to compare the reported outcomes in the study results, and we searched for the trial registration. We looked for the registry number in the article itself, and if it was not present, we searched on specific registry websites, including the following: “clinicaltrials.gov,” “anzctr.org.au,” “chictr.org.cn,” and “thaiclinicaltrials.org”. If we found registration, we defined its validity as follows: the registration was defined as Prospective-Valid if the date of registry submission preceded the trial start date; otherwise, the registration was defined as Retrospective-Invalid. If we did not retrieve a registration, we contacted the corresponding authors to check for registry availability. If we did not receive a response within one month of the initial contact, we assessed the reporting against the published article methods section. This process is described in Figure 1.

Secondly, we assessed how each of the outcomes (nausea, vomiting. or both) was described in both the study registry and article, using the factors proposed by Saldanha et al. for a “well-reported outcome”: (1) Type of outcome: Primary Outcome (PO) or Secondary Outcome (SO); (2) Domain: CIN (Chemotherapy-Induced Nausea), CIV (Chemotherapy-Induced Vomiting), or CINV; (3) Specific measurement: the technique or instrument used for outcome measurement; (4) Specific metric: the format of outcome data used for analysis, such as endpoint value or change from baseline. If not stated, we judged as “unclear/not reported”; (5) Type of data: dichotomous, continuous, discrete, rate, or ordinal; (6) Methods of aggregation: how data were summarized, such as mean, percentage, or proportion; (7) Timepoint unit and time: time unit of evaluation and measurement timepoint used for analysis.

Finally, we checked for discrepancies between the registry and article for each of the seven domains. When a registration was not found, outcomes were assessed by comparing the results and methods sections in the paper. If the description was unclear or absent, we defined the domain as “incomplete” and judged the discrepancy as “unclear”.

These review results are reported according to the PRISMA reporting checklist [10].

## 3. Results

**Figure 2:**
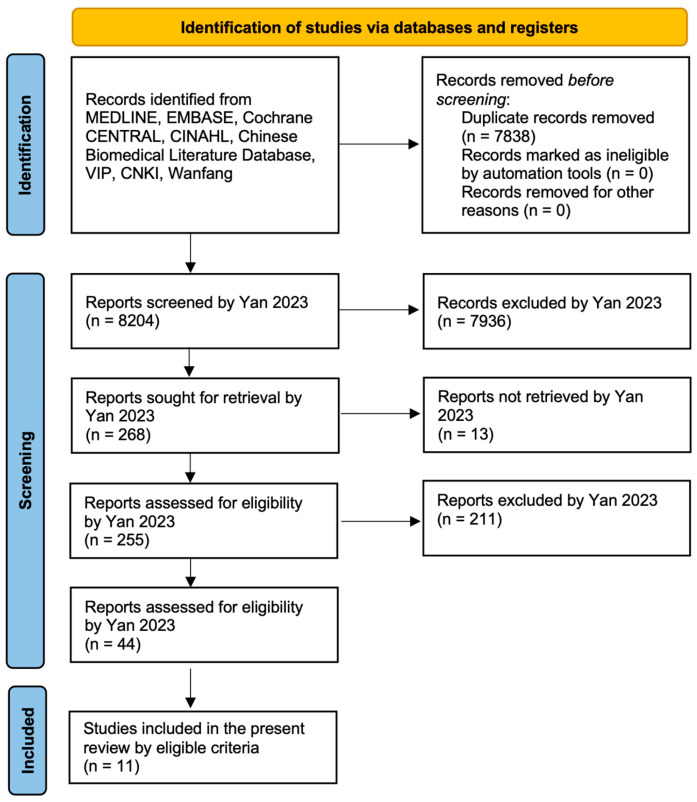
Flow diagram in accordance with the PRISMA guidelines. Identification and screening criteria were reported by Yan et al. [8].

We evaluated a total of 11 RCTs [11,12,13,14,15,16,17,18,19,20,21], published in English between 1987 and 2019 (Table 1). The PRISMA flowchart of the selection procedures of the studies is reported in Figure 2. Four studies were conducted in Europe (Germany and Ireland), one in the United States (California), four in Asia (China, Indonesia, and Thailand), and two in Australia. The mean sample size was 91.1, ranging from 10 to 220 patients. We found the study registration of four studies (36.4%), of which two were prospective and valid and two were retrospective and therefore invalid.

Table 2 provides a comprehensive overview of the outcomes reported in each of the eleven randomized controlled trials included in our review. The majority of studies (*n* = 9) reported on both nausea and vomiting as a combined outcome (CINV), while two studies focused solely on nausea or vomiting. Most outcomes were categorized as primary, although two studies included non-primary outcomes as well. A wide range of measurement instruments were employed, including validated tools such as the Multinational Association of Supportive Care in Cancer (MASCC, Aurora, ON, Canada) antiemetic tool [23], the Functional Living Index–Emesis (FLIE) [24], and the Common Terminology Criteria for Adverse Events (CTCAE) grading system, as well as study-specific or partially defined symptom counts and grading systems. The most commonly used outcome metrics were endpoint values (*n* = 5), change from baseline (*n* = 3), and values at a specific timepoint (*n* = 3). All studies reported discrete data types, though the methods of aggregation varied, with some reporting total frequencies, and others using measures of central tendency such as mean, median, or interquartile ranges. Timepoints for outcome assessment were heterogeneous and ranged from hours to months, with most studies reporting data on a daily basis. However, some studies did not clearly define the time unit used for measurement.

**Figure 3:**
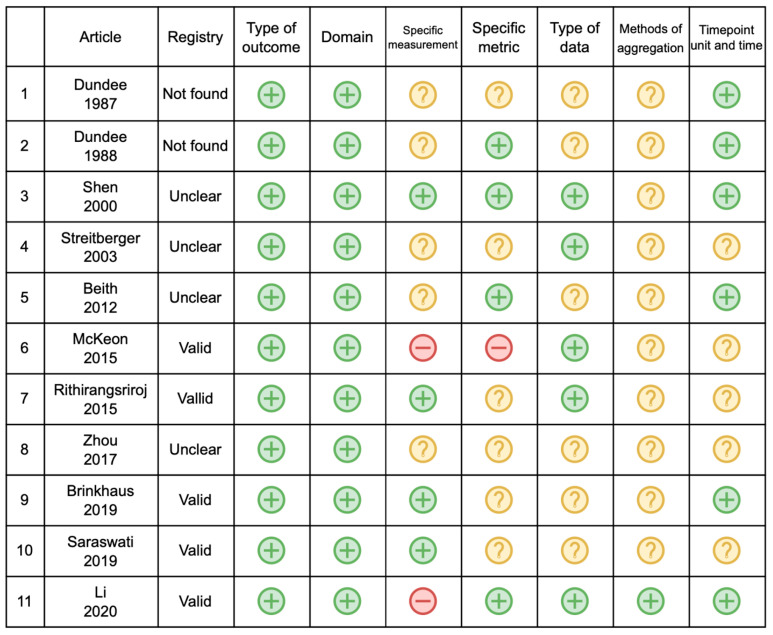
Discrepancies between the plan and the observed outcomes [11,12,13,14,15,17,18,19,20,21,22]. Green = no discrepancies between registered and reported outcomes; Yellow = domain unclear or not assessable due to missing or incomplete information; Red = discrepancy between registered and reported outcomes.

**Figure 4:**
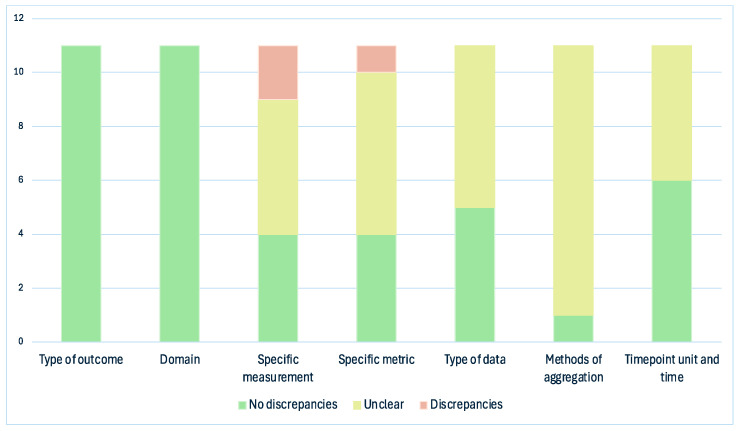
Discrepancies distribution. Green = no discrepancies between registered and reported outcomes; Yellow = domain unclear or not assessable due to missing or incomplete information; Red = discrepancy between registered and reported outcomes.

Figure 3 and Figure 4 summarize the discrepancies between the registry and article for each of the seven domains. Only in one study could all the domains be evaluated. In the other cases, the number of assessable domains went from two (*n* = 1) to six (*n* = 1).

No studies presented discrepancies concerning the type of outcome and domain. Regarding the specific measurement factor, four studies (36.4%) had no discrepancies, while for five studies, this was unclear. The remaining two studies had discrepancies in the measurement of outcomes. Li et al. [16] defined two specific measurements: the CTCAE grade, which was consistent between the registry and the article, and the second outcome, which was set in the registry as complete control of CINV and reported in the article as the variation of CINV degree. On the other hand, McKeon et al. [17] presented discrepancies in two secondary outcomes. The registry stated that the number of vomits would be measured, but the article reported a vomiting score with the Numeric Rating Scale (NRS). The other discrepant outcome was the nausea score, planned in the registry with the Visual Analogue Scale (VAS), while reported in the article with the NRS scale.

Concerning the specific metric factor, four studies (36.4%) had no discrepancies, where endpoint values were used as planned in the registry. The presence of discrepancies was unclear in six studies, as it was not specifically defined. McKeon et al. [17] presented a discrepancy, as the specific metric was changed for two secondary outcomes, nausea and vomiting: the registry defined these outcomes as endpoints, while in the published paper, both outcomes were reported as changes from the baseline. Regarding the type of data factor, five studies (45.5%) were consistent, while the remaining six were defined as unclear, as the item was often not described in the registry or in article methods.

The methods of aggregation were consistent in only one study, while in all the remaining studies, this item was not adequately defined. Concerning the timepoint unit and time, six studies (54.5%) were consistent, while for the remaining five studies, the rating was unclear.

## 4. Discussion

In our review on the quality of outcome reporting in RCTs investigating acupuncture for CINV, we found valid, prospective study registrations for only four studies (36.4%), showing how selective outcome reporting is a real issue in this research area. Additionally, none of the included studies provided complete and consistent reporting of the study outcomes of interest (chemotherapy-induced nausea or vomiting). The description of the outcomes was frequently incomplete, both in the registration and reporting phases, and we identified discrepancies between the registration and the published article. Discordant data were observed for the specific metric and specific measurement items. Moreover, the data reveal a marked heterogeneity in how outcomes were defined and reported. While most studies relied on discrete data types and reported total counts or central tendency measures (means or medians), there were notable inconsistencies in the instruments used—ranging from validated scales like the MASCC antiemetic tool to less standardized measures such as number of symptoms or severity grades. In several studies, outcome domains and metrics were partially or poorly reported. These inconsistencies highlight the lack of standardization in outcome reporting for acupuncture trials targeting chemotherapy-induced nausea and vomiting, further underscoring the risk of selective reporting bias.

Inconsistencies between registered and published primary outcomes of clinical trials have been identified in oncology clinical trials [6,7] and also in previous meta-epidemiological research. Walker et al. [25] found discrepancies in the primary outcome between registry and article in 33% of studies published in high-impact-factor journals, such as *JAMA* and *BMJ*. Chen et al. [26] concluded that 33.4% (130 of 389) of studies with clear primary outcomes prospectively described in a registry had at least one change in the primary outcome reported in the manuscript, with most of the changes (66 out of 130) either not reported or omitting the primary outcome.

A strength of our study is that we performed an assessment of outcome-reporting bias in the under-researched areas of acupuncture research and Patient-Reported Outcome Measures (PROMs), which are essential for studying the effects of Traditional Chinese Medicine (TCM) treatments. Numerous surveys on acupuncture use have shown that patients often seek it for pragmatic reasons [27,28], including relief from symptoms such as pain, anxiety, and depression; relief from side effects of medical treatments; or to improve and maintain general physical and mental health. All of these reasons can be assessed via self-report, making PROMs of primary interest in acupuncture studies [22,29]. While Western medicine studies often relegate patient-reported outcomes to a secondary status behind objective indicators, in acupuncture studies, they are typically of primary interest. Therefore, it is vital that PROMs are well-defined and reported in TCM studies. Our study, while demonstrating weaknesses in a specific area of integrative oncology research, will serve as a stimulus to improve the methodological quality of the entire field of study. From what we have observed in our study, a recommendation about a core set of PROMs to be consistently recorded and measured in acupuncture RCTs for CINV may be issued. Even if published after the last date of inception of our search, the MASCC-ESMO guidelines [30] mentioning validated tools such as the FLIE or the MASCC can be considered as a source of such recommendation. A valid, standardized, and reproducible measurement of CINV is of utmost importance in this crucial field of supportive oncology.

One way to reduce selective outcome-reporting bias is to require all investigators to register details of their trial in a publicly accessible registry before the trial starts and to adopt the World Health Organization Minimal Registration Dataset. This ensures that compulsory fields are completed when a trial is registered (WHO Trial Registration Data Set Version 1.2.1). Clinical trial registries gained increased interest from the 1970s due to the need to enhance patient enrollment in clinical trials and to reduce the possibility of bias in reporting trial results. In 2004, the International Committee of Medical Journal Editors (ICMJE) initiated a policy requiring investigators to register their trials in a clinical trial registry before participant enrollment begins as a condition of publication in one of their journals. The WHO developed the International Standards for Clinical Trial Registries (ISCTR), which lists the minimum standards that registries should adopt to ensure a basic quality of data and accessibility. Unfortunately, these standards aren’t often met, and missing information is often present.

Furthermore, registration may be omitted or post-dated, or the information provided in the trial registration may not correlate with the trial publication, leading to publication and outcome reporting bias. Both biases threaten the validity of evidence-based medicine, as clinicians only have access to the results that researchers choose to publish. Strict, comprehensive registration of trials at their outset allows the differences between what was originally planned in the study and what is eventually published to be seen, allowing critical evaluation of the trial and minimizing these two sources of bias. This is especially important in the TCM field, where recognition of effective treatments not considered by Western medicine cannot be hampered by methodological weaknesses in studies.

The main limitation of our study is the limited number of studies included; this evidence reflects a deliberate methodological choice related to registry accessibility and consistency in outcome extraction. While our study may be limited in its small sample size and inclusion of studies written only in English, excluding 21 studies from China due to the language, it can serve as a blueprint for future investigations, considering TCM treatments other than acupuncture.

**Figure 5:**
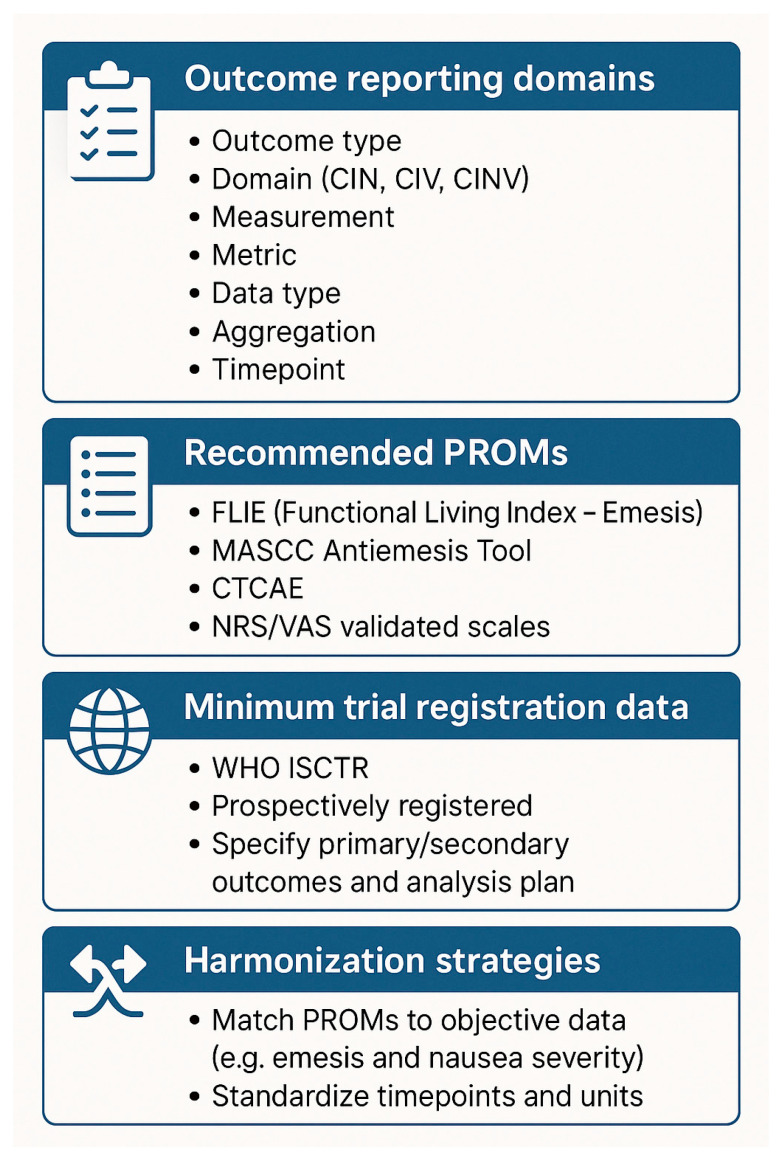
Visual reporting framework summarizing the key components to enhance methodological guidance for future research, we propose a visual framework.

The findings of our study have relevant clinical implications. Incomplete or selectively reported outcomes in acupuncture trials for CINV can mislead clinicians about the true effectiveness of these interventions, especially when outcome definitions, metrics, and timepoints are not standardized. Without reliable, transparent reporting, it may become difficult for oncologists and integrative medicine practitioners to make evidence-informed decisions or to compare interventions across studies. Changing an outcome metric or failing to register it in advance can make cherry-picking results easier, introducing bias toward positive findings and undermining the credibility of scientific research [31]. To improve the methodological transparency and clinical applicability of future acupuncture trials, we recommend the following: (i) a prospective and detailed trial registration should be mandatory, including full specification of all seven outcome components (as outlined by Saldanha et al.) at the protocol level; (ii) the adoption of reporting guidelines, such as the CONSORT for Non-Pharmacologic Treatments; and (iii) researchers should prioritize consistency between registry, protocol, and final publication and clearly justify any changes to outcome definitions. However, despite the methodological limitations we reported in the relevant literature, acupuncture for the control of CINV is anyway recommended in the MASCC-ESMO guidelines [30]: clinicians shall suggest this intervention for acute vomiting occurring the first day of the treatment course, and this TCM treatment must become standard care in supportive oncology services. To enhance methodological guidance for future research, we propose a visual framework (Figure 5) summarizing key components for designing and reporting acupuncture trials for CINV. This tool may assist researchers in aligning outcome measures, patient-reported metrics, and trial registration standards, avoiding selective reporting, and ultimately supporting evidence harmonization [32].

## 5. Conclusions

In conclusion, our study has shown the difficulties in finding complete outcome descriptions and reporting in studies evaluating the effects of acupuncture on chemotherapy-induced nausea and vomiting (CINV). It is fundamental for researchers to determine these outcomes before proceeding with their research and to register them properly in order to produce solid evidence. Improving the transparency of outcome reporting in acupuncture research will not only strengthen the credibility of published results but will also foster trust among clinicians and patients exploring integrative oncology options.

## Figures and Tables

**Table 1:** Study characteristics and registration details.

First Author	Year	Country	Study Design	Sample Size	Study ID	Study Registration	1st Recruitment Date
Dundee [15]	1987	Ireland	Randomized trial	10	Not found	Not found	Not found
Dundee [14]	1988	Ireland	Randomized trial	20	Not found	Not found	Not found
Shen [19]	2000	California	Three-arm, parallel-group, randomized controlled trial	104	Not found	Unclear	Not applicable
Streitberger [20]	2003	Germany	Randomized placebo-controlled single-blind trial	220	Not found	Unclear	Not applicable
Beith [11]	2012	Australia	Randomized controlled pilot trial	32	Not found	Unclear	Not applicable
McKeon [17]	2015	Australia	Single-centre, pilot, randomised trial	153	ACTRN 126090010554202	09/12/09	April 2009
Rithirangsriroj [13]	2015	Thailand	Randomized trial, crossover study	70	TCTR 20121105001	16/05/13	May 2013
Zhou [21]	2017	China	Randomized controlled trial	56	Not found	Unclear	Not applicable
Brinkhaus [12]	2019	Germany	Randomized pragmatic trial	150	NCT 01727362	16/11/12	October 2012
Saraswati [18]	2019	Indonesia	Randomized, experimental clinical study	53	Not found	Unclear	Not applicable
Li [22]	2020	China	Multi-center, single-blind, randomized, sham-controlled clinical trial	134	NCT 02369107	23/02/15	March 2015

**Table 2:** Study outcomes.

First Author, Year	Outcome	Type	Domain	Measurement	Specific Metric	Type of Data	Method of Aggregation	Timepoints Unit
Dundee, 1987 [15]	Nausea and vomit	PO	CINV	Four grades of sickness	Not reported	Discrete	Grade of severity	Not reported
Dundee, 1988 [14]	Nausea and vomit	PO	CINV	Number of subjects with symptoms	Endpoint	Discrete	Total number of patients	Hours
Shen, 2000 [19]	Vomit	PO	CIN	Total number of emesis episodes	Value at a timepoint	Discrete	Total, median, range, mean, percentiles	Days
Streitberger, 2003 [20]	Nausea and vomit	PO and non -PO	CINV	Number of patients, frequency of vomit, duration of nausea	Value at a timepoint	Discrete	Total number of patients	Days
Beit, 2012 [11]	Nausea and vomit	PO	CINV	Multinational Association of Supportive Care in Cancer (MASCC) antiemetic tool	Endpoint	Discrete	Total number of patients and percent age	Days
McKeon, 2015 [17]	Nausea and vomit	PO	CINV	Function Living Index—Emesis, Vomit and Nausea NRS	Change from baseline and Endpoint	Discrete	Median and IQR	Days
Rithirangs riroj, 2015 [13]	Nausea and vomit	PO	CINV	Number of emesis and nausea degree	Endpoint	Discrete	Median and range	Not reported
Zhou, 2017 [21]	Nausea and vomit	PO	CINV	Frequency of vomit, duration of nausea	Value at a timepoint	Value at a timepoint	Mean	Days
Brinkhaus, 2019 [12]	Nausea	Non -PO	CIN	Single item FACT B	Change from the baseline	Discrete	Mean and 95% CI	Months
Saraswati, 2019 [18]	Nausea and vomit	Non -PO	CINV	Modified Rhodes Index of Nausea and Vomiting	Not reported	Not reported	Not reported	Not reported
Li, 2020 [22]	Nausea and vomit	PO	CINV	CTCAE grade	Change from baseline	Discrete	Mean + SD	Days

PO, primary outcome; non-PO, non-primary outcome; CINV, chemotherapy-induced nausea and vomit; CI, confidence interval; SD, standard deviation; IQR, interquartile range.

## Data Availability

The data underlying this article will be shared by the corresponding author upon reasonable request.

## Supplementary Materials

The following supporting information can be downloaded at https://www.mdpi.com/article/10.3390/curroncol32080462/s1, Supplementary File. Search Strategy.

## Abbreviations

The following abbreviations are used in this manuscript:

RCTs	Randomized Controlled Trials
TCM	Traditional Chinese Medicine
CINV	Chemotherapy-Induced Nausea and Vomiting
CIN	Chemotherapy-Induced Nausea
CIV	Chemotherapy-Induced Vomiting
VAS	Visual Analogue Scale
PROMs	Patient-Reported Outcome Measures
IS-CTR	International Standards for Clinical Trial Registries
MASCC	Multinational Association of Supportive Care in Cancer

## References

[1] Nankervis H., Baibergenova A., Williams H.C., Thomas K.S. (2012). Prospective Registration and Outcome-Reporting Bias in Randomized Controlled Trials of Eczema Treatments: A Systematic Review. J. Investig. Dermatol..

[2] Kirkham J.J., Dwan K.M., Altman D.G., Gamble C., Dodd S., Smyth R., Williamson P.R. (2010). The impact of outcome reporting bias in randomised controlled trials on a cohort of systematic reviews. BMJ.

[3] Boutron I., Page M.J., Higgins J.P.T., Altman D.G., Lundh A., Hróbjartsson A., Higgins J.P.T., Thomas J., Chandler J., Cumpston M., Li T., Page M.J., Welch V.A. (2023). Chapter 7: Considering bias and conflicts of interest among the included studies. Cochrane Handbook for Systematic Reviews of Interventions Version 6.4 (Updated August 2023).

[4] Liu J.P., Han M., Li X.X., Mu Y.-J., Lewith G., Wang Y.-Y., Witt C.M., Yang G.-Y., Manheimer E., Snellingen T. (2013). Prospective registration, bias risk and outcome-reporting bias in randomised clinical trials of traditional Chinese medicine: An empirical methodological study. BMJ Open.

[5] Su C.X., Han M., Ren J., Li W.Y., Yue S.J., Hao Y.F., Liu J.P. (2015). Empirical evidence for outcome reporting bias in randomized clinical trials of acupuncture: Comparison of registered records and subsequent publications. Trials.

[6] Vera-Badillo F.E., Napoleone M., Krzyzanowska M.K., Alibhai S.M., Chan A.W., Ocana A., Seruga B., Templeton A.J., Amir E., Tannock I.F. (2016). Bias in reporting of randomised clinical trials in oncology. Eur. J. Cancer.

[7] Singh B., Fairman C.M., Christensen J.F., Bolam K.A., Twomey R., Nunan D., Lahart I.M. (2021). Outcome Reporting bias in Exercise Oncology trials (OREO): A cross-sectional study. medRxiv.

[8] Yan Y., López-Alcalde J., Zhang L., Siebenhüner A.R., Witt C.M., Barth J. (2023). Acupuncture for the prevention of chemotherapy-induced nausea and vomiting in cancer patients: A systematic review and meta-analysis. Cancer Med..

[9] Saldanha I.J., Dickersin K., Wang X., Li T. (2014). Outcomes in Cochrane Systematic Reviews Addressing Four Common Eye Conditions: An Evaluation of Completeness and Comparability. PLoS ONE.

[10] Page M.J., McKenzie J.E., Bossuyt P.M., Boutron I., Hoffmann T.C., Mulrow C.D., Shamseer L., Tetzlaff J.M., Akl E.A., Brennan S.E. (2021). The PRISMA 2020 statement: An updated guideline for reporting systematic reviews. BMJ.

[11] Beith J.M., Oh B., Chatfield M.D., Davis E., Venkateswaran R. (2012). Electroacupuncture for Nausea, Vomiting, and Myelosuppression in Women Receiving Adjuvant Chemotherapy for Early Breast Cancer: A Randomized Controlled Pilot Trial. Med. Acupunct..

[12] Brinkhaus B., Kirschbaum B., Stöckigt B., Binting S., Roll S., Carstensen M., Witt C.M. (2019). Prophylactic acupuncture treatment during chemotherapy with breast cancer: A randomized pragmatic trial with a retrospective nested qualitative study. Breast Cancer Res. Treat..

[13] Rithirangsriroj K., Manchana T., Akkayagorn L. (2015). Efficacy of acupuncture in prevention of delayed chemotherapy induced nausea and vomiting in gynecologic cancer patients. Gynecol. Oncol..

[14] Dundee J.W., Ghaly R.G., Fitzpatrick K.T.J. (1988). Randomised comparison ofthe anti-emetic effects of metoclopramide and electro acupuncture in cancer chemotherapy. Br. J. Clin. Pharmacol..

[15] Dundee J.W., Ghaly R.G., Fitzpatrick K.T.J., Lynch G.A., Abram W.P. (1987). Acupuncture to prevent cisplatin-associated vomiting. Lancet.

[16] Li Q.W., Yu M.W., Wang X.M., Yang G.-W., Wang H., Zhang C.-X., Xue N., Xu W.-R., Zhang Y., Cheng P.-Y. (2020). Efficacy of acupuncture in the prevention and treatment of chemotherapy-induced nausea and vomiting in patients with advanced cancer: A multi-center, single-blind, randomized, sham-controlled clinical research. Chin Med..

[17] McKeon C., Smith C.A., Gibbons K., Hardy J., Haugstetter C., Anderson H. (2015). Ea versus Sham Acupuncture and no Acupuncture for the Control of Acute and Delayed Chemotherapy-Induced Nausea and Vomiting: A Pilot Study. Acupunct. Med..

[18] Saraswati W., Dahlan E.G., Saputra K., Sutrisno T.C. (2019). Effect of Electroacupuncture on Natural-Killer Cells and Tumor Size in Patients with Cervical Squamous-Cell Carcinoma: A Randomized Controlled Trial. Med. Acupunct..

[19] Shen J., Wenger N., Glaspy J., Hays R.D., Albert P.S., Choi C., Schkelle P.G. (2000). Electroacupuncture for Control of Myeloablative Chemotherapy–Induced Emesis: A Randomized Controlled Trial. JAMA.

[20] Streitberger K., Friedrich-Rust M., Bardenheuer H., Unnebrink K., Windeler J., Goldschmidt H., Egerer G. (2003). Effect of acupuncture compared with placebo-acupuncture at P6 as additional antiemetic prophylaxis in high-dose chemotherapy and autologous peripheral blood stem cell transplantation: A randomized controlled single-blind trial. Clin. Cancer Res..

[21] Zhou J., Fang L., Wu W.Y., He F., Zhang X.L., Zhou X., Xiong Z.J. (2017). The Effect of Acupuncture on Chemotherapy-Associated Gastrointestinal Symptoms in Gastric Cancer. Curr. Oncol..

[22] Li T., Park S.B., Battaglini E., King M.T., Kiernan M.C., Goldstein D., Rutherford C. (2022). Assessing chemotherapy-induced peripheral neuropathy with patient reported outcome measures: A systematic review of measurement properties and considerations for future use. Qual. Life Res..

[23] Molassiotis A., Coventry P.A., Stricker C.T., Clements C., Eaby B., Velders L., Rittenberg C., Gralla R.J. (2007). Validation and psychometric assessment of a short clinical scale to measure chemotherapy-induced nausea and vomiting: The MASCC antiemesis tool. J. Pain Symptom Manag..

[24] Decker G.M., DeMeyer E.S., Kisko D.L. (2006). Measuring the maintenance of daily life activities using the functional living index-emesis (FLIE) in patients receiving moderately emetogenic chemotherapy. J. Support. Oncol..

[25] Walker K.F., Stevenson G., Thornton J.G. (2014). Discrepancies between registration and publication of randomised controlled trials: An observational study. JRSM Open.

[26] Chen T., Li C., Qin R., Wang Y., Yu D., Dodd J., Wang D., Cornelius V. (2019). Comparison of Clinical Trial Changes in Primary Outcome and Reported Intervention Effect Size Between Trial Registration and Publication. JAMA Netw. Open.

[27] Chow Y.M., Tong C.K., Chutatape T.T., Seah C.N., Cui S.L., Tan K.H., Chan D.X. (2022). Survey of patients’ perspectives on the use of acupuncture as a complementary treatment for chronic pain. Bali J. Anesthesiol..

[28] Greville-Harris M., Hughes J., Lewith G., Liossi C., White P., Graham C.A., Bishop F. (2016). Assessing knowledge about acupuncture: A survey of people with back pain in the UK. Complement. Ther. Med..

[29] Vernooij M., Marcelissen F. (2017). Measuring patient reported outcomes of acupuncture treatment on pain patients’ health status. Complement. Ther. Clin. Pract..

[30] Herrstedt J., Clark-Snow R., Ruhlmann C.H., Jordan K., Scotté F. (2023). MASCC/ESMO antiemetic guidelines: Introduction to the 2023 guidelines update. Support. Care Cancer.

[31] Odone A., Sgueglia A.C., Bertuccio P., Vecchio R., Meloni A., Gianfredi V., Traverso L., Gaeta M., Vigezzi G.P. (2024). “Leo&Giulia standing for public health”: An animated series to promote the values of public health among school-aged children. Best practices and field-trial protocol. Ann. Ig..

[32] Mayo-Wilson E., Li T., Fusco N., Bertizzolo L., Canner J.K., Cowley T., Doshi P., Ehmsen J., Gresham G., Guo N. (2017). Cherry-picking by trialists and meta-analysts can drive conclusions about intervention efficacy. J. Clin. Epidemiol..

