# Correction: Penati et al. High Outcome-Reporting Bias in Randomized-Controlled Trials of Acupuncture for Cancer Chemotherapy-Induced Nausea and Vomiting: A Systematic Review and Meta-Epidemiological Study. *Curr. Oncol.* 2025, *32*, 462

**DOI:** 10.3390/curroncol33030131

**Published:** 2026-02-24

**Authors:** Rachele Penati, Riccardo Vecchio, Roberto Gatto, Anna Odone, Silvia Deandrea

**Affiliations:** 1 Villa Beretta Rehabilitation Center, Valduce Hospital, 23845 Costa Masnaga, Italy; 2 Centro Studi So Wen ETS, 20146 Milano, Italy; 3 Department of Public Health, Experimental and Forensic Medicine, University of Pavia, 27100 Pavia, Italy; 4 Medical Direction, Fondazione IRCCS Policlinico San Matteo, 27100 Pavia, Italy

The authors inadvertently omitted to indicate a second affiliation of Anna Odone in the original publication [1]. The authors state that the scientific conclusions are unaffected. This correction was approved by the Academic Editor. The original publication has also been updated.

## References

[1] Penati R., Vecchio R., Gatto R., Odone A., Deandrea S. (2025). High Outcome-Reporting Bias in Randomized-Controlled Trials of Acupuncture for Cancer Chemotherapy-Induced Nausea and Vomiting: A Systematic Review and Meta-Epidemiological Study. Curr. Oncol..

