# Supplementary material for: High Outcome-Reporting Bias in Randomized-Controlled Trials of Acupuncture for Cancer Chemotherapy-Induced Nausea and Vomiting: A Systematic Review and Meta-Epidemiological Study
Source: Curr Oncol. 2025 Aug 15;32(8):462. doi: 10.3390/curroncol32080462 (PMC12385644; doi:10.3390/curroncol32080462)
Supplement: Supplementary file 1 [file curroncol-32-00462-s001.zip › curroncol-3725153-supplementary.pdf]

Supplementary Materials I. Search Strategy

Only studies written in English were included

MEDLINE (via PubMed)

Search	Query
#23	Search: #11 AND #22
#22	Search: #20 NOT #21
#21	Search: animals [mh] NOT humans [mh]
#20	Search: #12 OR #13 OR #14 OR #15 OR #16 OR #17 OR #18 OR #19
#19	Search: groups[Title/Abstract]
#18	Search: trial[Title/Abstract]
#17	Search: randomly[Title/Abstract]
#16	Search: drug therapy[MeSH Subheading]
#15	Search: placebo[Title/Abstract]
#14	Search: randomized[Title/Abstract]
#13	Search: controlled clinical trial[Publication Type]
#12	Search: randomized controlled trial[Publication Type]
#11	Search: #3 AND #10
#10	Search: #8 OR #9

Search	Query
#9	Search: "Neoplasms"[Mesh]
#8	Search: #4 OR #5 OR #6 OR #7
#7	Search: Mammacarcinom*[Text Word] OR Maltoma*[Text Word] OR Masculinovoblastoma*[Text Word] OR Mastocytoma*[Text Word] OR Medulloblastoma*[Text Word] OR Medullocytoma*[Text Word] OR Medulloepithelioma*[Text Word] OR Medullomyoblastoma*[Text Word] OR Melanoacanthoma*[Text Word] OR Melanoameloblastoma*[Text Word] ORMelanocytoma*[Text Word] OR Melanoma*[Text Word] OR Meningioma*[Text Word] OR Mesenchymoma*[Text Word] OR Mesonephroma*[Text Word] OR Mesothelioma*[Text Word] OR Microcarcinoma*[Text Word] OR Microglioma*[Text Word] OR Micrometastas*[Text Word] OR Myelolipoma*[Text Word] OR [Text Word] OR Myeloma*[Text Word] OR Myoepithelioma*[Text Word] OR Myoblastoma*[Text Word] OR Myoepithelioma*[Text Word] OR Myofibroblastoma*[Text Word] OR Myofibroma*[Text Word] OR Myofibrosarcoma*[Text Word] OR Myolipoma*[Text Word] OR Myoma*[Text Word] OR Myopericytoma*[Text Word] OR Myosarcoma*[Text Word] OR Myxofibroma*[Text Word] OR Myxolipoma*[Text Word] OR Myxoliposarcoma*[Text Word] OR Myxoma*[Text Word] OR Naevus[Text Word] OR Nephroblastoma*[Text Word] OR Nephroma*[Text Word] OR Neurilemmoma*[Text Word] OR Neurilemoma*[Text Word] OR Neuroblastoma*[Text Word] OR Neurocytoma*[Text Word] OR Neuroepithelioma*[Text Word] OR Neurofibroma*[Text Word] OR Neurofibrosarcoma*[Text Word] OR Neurolipocytoma*[Text Word] OR Neuroma*[Text Word] OR Neuronevus[Text Word] OR Neurothekeoma*[Text Word] OR Nevus[Text Word] OR nonhodgkin[Text Word] OR Non-Hodgkin[Text Word] OR Nonseminoma*[Text Word] OR Odontoameloblastoma*[Text Word] OR Odontoma*[Text Word] OR Oligoastrocytoma*[Text Word] OR Oligodendroglioma*[Text Word] OR Oncocytoma*[Text Word] OR Orchioblastoma*[Text Word] OR Osteoblastoma*[Text Word] OR Osteochondroma*[Text Word] OR Osteochondrosarcoma*[Text Word] OR Osteoclastoma*[Text Word] OR Osteofibrosarcoma*[Text Word] OR Osteoma*[Text Word] OR Osteosarcoma*[Text Word] OR Pancreatoblastoma*[Text Word] OR Parachordoma*[Text Word] OR Paraganglioma*[Text Word] OR Paraneoplastic[TextWord] OR Perineurioma*[Text Word] OR Phaeochromocytoma*[Text Word] OR Pheochromoblastoma*[Text Word] OR Pheochromocytoma*[Text Word] OR Pilomatricoma*[Text Word] OR Pilomatrixoma*[Text Word] OR Pinealblastoma*[Text Word] OR Pinealoma*[Text Word] OR Pineoblastoma*[Text Word] OR Pineocytoma*[Text Word] OR Plasmacytom*[Text Word] OR

Search	Query
	Pneumoblastoma*[Text Word] OR Pneumocytoma*[Text Word] OR Polyembroma*[Text Word] OR Polyhistioma*[Text Word] OR Porocarcinoma*[Text Word] OR Poroma*[Text Word] OR Reninoma*[Text Word] OR Reticuloendothelioma*[Text Word] OR Reticulohistiocytoma*[Text Word] OR Retinoblastoma*[Text Word] OR Rhabdomyoma*[Text Word] OR Rhabdomyosarcoma*[Text Word] OR Rhabdosarcoma*[Text Word] OR Sarcoid*[Text Word] OR Sarcoma*[Text Word] OR Schwannoma*[Text Word] OR Seminoma*[Text Word] OR Somatostaminoma*[Text Word] OR Somatotropinoma*[Text Word] OR Spermatocynoma*[Text Word] OR Spiradenoma*[Text Word] OR Spongioblastoma*[Text Word] OR Steatocystoma*[Text Word] OR Subependymoma*[Text Word] OR Syringadenoma*[Text Word] OR Syringoma*[Text Word] OR Teratocarcinoma*[Text Word] OR Teratoma*[Text Word] OR Thecoma*[Text Word]OR Thymolipoma*[Text Word] OR Thymoma*[Text Word] OR Trichilemmoma*[Text Word] OR Trichoadenoma*[Text Word] OR Trichoblastoma*[Text Word] OR Trichodiscoma*[Text Word] OR Trichoepithelioma*[Text Word] OR Trichofolliculoma*[Text Word] OR Tricholemmoma*[Text Word] OR Vipoma*[Text Word] OR Waldenstrom*[Text Word] OR Xanthoastrocytoma*[Text Word] OR Xanthofibroma*[Text Word] OR Xanthogranuloma*[Text Word] OR Xanthoma*[Text Word] OR Xanthosarcoma*[Text Word]
#6	Search: Dentinoma*[Text Word] OR Dermatofibroma*[Text Word] OR Dermatofibrosarcoma*[Text Word] OR Dictyoma*[Text Word] OR Dysgerminoma*[Text Word] OR Dyskeratoma*[Text Word] OR Dysplasia*[Text Word] OR Dysplastic*[Text Word] OR Ectomesenchymoma*[Text Word] OR Elastofibroma*[Text Word] OR Enchondroma*[Text Word]OR Endothelioma*[Text Word] OR Ependymoblastoma*[Text Word] OR Ependymoma*[Text Word] OR Epitheliom*[Text Word] OR Erythroleukaemia*[Text Word] OR Erythroleukemia*[Text Word] OR Esthesioneuroblastoma*[Text Word] OR Esthesioneuroepithelioma*[Text Word] OR Fibroadenoma*[Text Word] OR Fibroadenosarcoma*[Text Word] OR Fibroelastoma*[Text Word] OR Fibroepithelioma*[Text Word] OR Fibrofolliculoma*[Text Word] OR Fibrolipoma*[Text Word] OR Fibroliposarcoma*[Text Word] OR Fibroma*[Text Word] OR Fibromyxoma*[Text Word] OR Fibroodontoma*[Text Word] OR Fibrosarcoma*[Text Word] OR Fibro- sarcoma*[Text Word] OR Fibrothecoma*[Text Word]OR Fibroxanthoma*[Text Word] OR Fibroxanthosarcoma*[Text Word] OR Ganglioblastoma*[Text Word] OR Gangliocytoma*[Text Word] OR Ganglioglioma*[Text Word] OR Ganglioneuroblastoma*[Text Word] OR Ganglioneurofibroma*[Text Word] OR Ganglioneuroma*[Text Word] OR Gastrinoma*[Text Word] OR Germinoma*[Text Word] OR

Search	Query
	Glioblastoma*[Text Word] OR Gliofibroma*[Text Word] OR Glioma*[Text Word] OR Glioneuroma*[Text Word] OR Gliosarcoma*[Text Word] OR Glomangioma*[Text Word] OR Glomangiomyoma*[Text Word] OR Glomangiosarcoma*[Text Word] OR Glucagonoma*[Text Word] OR Gonadoblastom*[Text Word] OR Gonocytoma*[Text Word] OR Granuloma*[Text Word] OR Gynandroblastoma*[Text Word] OR Haemangioblastoma*[Text Word] OR Hemangioblastoma*[Text Word] OR Hemangiom*[Text Word] OR Haemangiom*[Text Word] OR Hemangiopericytom*[Text Word] OR Haemangiopericytom*[Text Word] OR Hemangioendothelioma*[Text Word] OR Haemangioendothelioma*[Text Word] OR Hemangioendotheliosarcoma*[Text Word] OR Haemangioendotheliosarcoma*[Text Word] OR Hemangiopericytoma*[Text Word] OR Haemangiopericytoma*[Text Word] OR Hemangiosarcoma*[Text Word] OR Haemangiosarcoma*[Text Word] OR Hepatoblastom*[Text Word] OR Hepatocarcinoma*[Text Word] OR Hepatocholangiocarcinoma*[Text Word] OR Hepatoma*[Text Word] OR Hibernoma*[Text Word] OR Hidradenoma*[Text Word] OR Histiocytoma*[Text Word] OR Hodgkin*[Text Word] OR Hydradenoma*[Text Word] OR Hypernephroma*[Text Word] OR Immunocytoma*[Text Word] ORKeratoacanthoma*[Text Word] OR Leiomyoblastoma*[Text Word] OR Leiomyofibroma*[Text Word] OR Leiomyoma*[Text Word] OR Leiomyosarcoma*[Text Word] OR Leukemia*[Text Word] OR Leukaemia*[Text Word] OR Leukoplakia*[Text Word] OR Lipoadenoma*[Text Word] OR Lipoblastoma*[Text Word] OR Lipoma*[Text Word] OR Liposacoma*[Text Word] OR Lymphom*[Text Word] OR Lymphangioendothelioma*[Text Word] OR Lymphangioma*[Text Word] OR Lymphangiomyoma*[Text Word] OR Lymphangiosarcoma*[Text Word] OR Lymphoepithelioma*[Text Word] OR Lymphoma*[Text Word] OR Lymphoproliferation[Text Word] OR Lymphoscintigraphy[Text Word]
#5	Search: Acanthoma*[Text Word] OR Adamantinoma*[Text Word] OR Adenoacanthoma*[Text Word] OR Adenocarcinoma*[Text Word] OR Adenoma*[Text Word] OR Adenolipoma*[Text Word] OR Adenolymphoma*[Text Word]OR adenomatous[Text Word] OR Adenomyoepithelioma*[Text Word] OR Adenomyoma*[Text Word] OR Adenosarcoma*[Text Word] OR Aesthesioneuroblastoma*[Text Word] OR Ameloblastoma*[Text Word] OR Amyloidosis[Text Word] OR Anaplasia[Text Word] OR Androblastoma*[Text Word] OR Angioblastoma*[Text Word] OR Angioendothelioma*[Text Word] OR Angiofibroma*[Text Word] OR Angiofibrosarcoma*[Text Word] OR Angiokeratoma* Angioleiomyoma*[Text Word] OR Angiolipoma*[Text Word] OR Angioma*[Text Word] OR

Search	Query
	Angiomyolipoma*[Text Word] OR Angiomyoma*[Text Word] OR Angiomyxoma*[Text Word] OR Angioreticuloma*[Text Word] OR Angiosarcoma*[Text Word] OR Apudoma*[Text Word] OR Argentaffinoma*[Text Word] OR Arrhenoblastoma*[Text Word] OR Astroblastoma*[Text Word] OR Astrocytoma*[Text Word] OR Astroglioma*[Text Word] OR Baltoma*[Text Word] OR Basiloma*[Text Word] OR Carcinosarcoma*[Text Word] OR Cavernoma*[Text Word] OR Cementoma*[Text Word] OR Ceruminoma*[Text Word] OR Chemodectoma*[Text Word] OR Cholangiocarcinoma*[Text Word] OR Cholangiohepatoma*[Text Word] OR Cholangioma*[Text Word] OR Cholangiosarcoma*[Text Word] OR Chondroblastoma*[Text Word] OR Chondro-Blastoma*[Text Word] OR Chondroma*[Text Word] OR Chondrosarcoma*[Text Word] OR Chondro-sarcoma*[Text Word] OR Chordoma[Text Word] OR Chorioadenoma*[Text Word] OR Chorioangioma*[Text Word] OR Choriocarcinoma*[Text Word] OR Chorioepithelioma*[Text Word] OR Choristoma*[Text Word] OR Cementoma*[Text Word] OR Collagenoma*[Text Word] OR Comedocarcinoma*[Text Word] OR Condyloma*[Text Word] OR Corticotropinoma*[Text Word] OR Craniopharyngioma*[Text Word] OR Cylindroma*[Text Word] OR Cyst?[Text Word] OR Cystadenocarcinoma*[Text Word] OR Cystadenofibroma*[Text Word] OR Cystadenoma*[Text Word] OR Cystoma*[Text Word] OR Cystosarcoma*[Text Word]
#4	Search: Cancer*[Text Word] OR Neoplas*[Text Word] OR Carcinom*[Text Word] OR Carcinogen*[Text Word] OR Malignan*[Text Word] OR Tumor*[Text Word] OR tumour*[Text Word] OR Anti-cancer[Text Word] OR Anticancer[TextWord] OR Anti-carcino*[Text Word] OR Anticarcino*[Text Word] OR Antitumor[Text Word] OR Antitumour[Text Word]OR Anti-tumor[Text Word] OR Anti-tumour[Text Word] OR Tumor-inhibit*[Text Word] OR Tumour-inhibit*[Text Word]OR Hyperplas*[Text Word] OR Metastas*[Text Word] OR Metastatic[Text Word] OR Metaplasia[Text Word] OR Oligometasta*[Text Word] OR Oligorecurren*[Text Word] OR Anticarcinogen*[Text Word] OR Antimutagenesis[Text Word] OR Anti-neoplastic[Text Word] OR Antineoplastic[Text Word] OR Antimetastatic[Text Word] OR Anti- metastatic[Text Word] OR Antioncogene[Text Word] OR Metastasectomy[Text Word] OR Oncolog*[Text Word] OR Oncogen*[Text Word] OR Oncolytic[Text Word] OR Lymph node excision*[Text Word] OR Sentinel lymph node*[Text Word] OR mammography[Text Word] OR mastectomy [Text Word]
#3	Search: #1 OR #2

Search	Query
#2	Search: (("Acupuncture"[Mesh] OR "Acupuncture Therapy"[Mesh]) OR "Acupressure"[Mesh])
#1	Search: "acupunct*"[Text Word] OR "electro acupunct*"[Text Word] OR "electroacupunct*"[Text Word] OR "electricstimulation*"[Text Word] OR "acupoint*"[Text Word] OR "acu point*"[Text Word] OR "acustimulation"[Text Word] OR "neiguan"[Text Word] OR "P6"[Text Word] OR "P- 6"[Text Word] OR "wristband"[Text Word] OR "wrist- band"[Text Word] OR "acupotom*"[Text Word] OR "auriculotherap*"[Text Word] OR "meridian*"[Text Word] OR "Ching Lo"[Text Word] OR "Jing Luo"[Text Word] OR "Jingluo"[Text Word] OR "Moxibustion"[Text Word] OR "Moxabustion"[Text Word] OR "pharmacoacupunct*"[Text Word] OR "pharmaco acupunct*"[Text Word] OR "pharmacopunct*"[Text Word] OR "Dequi"[Text Word] OR "acupress*"[Text Word] OR "shiatsu"[Text Word] OR "Tui Na"[Text Word] OR "zhi ya"[Text Word]
